# Residual symptoms and their associated factors among Thai patients with depression: a multihospital-based survey

**DOI:** 10.1186/s12991-022-00427-w

**Published:** 2022-12-16

**Authors:** Jarurin Pitanupong, Katti Sathaporn, Laddaporn Tepsuan

**Affiliations:** 1 Present Address: Department of Psychiatry, Faculty of Medicine, Prince of Songkla University, Hat Yai, Songkhla, 90110 Thailand; 2 Songkhla Hospital, Mueang Songkhla, Songkhla, 90110 Thailand

**Keywords:** Antidepressant, Depression, Expectation, Impact, Residual

## Abstract

**Background:**

Depression is a common and debilitating disease, and even residual symptoms of depression can cause significant functional impairment. To achieve normal functioning, residual symptoms should also be identified and targeted by a competent treatment strategy. Thus, this study aimed to examine residual symptoms of depression and their associated factors among patients with depression.

**Methods:**

A cross-sectional study surveyed Thai patients with depression at two psychiatric outpatient clinics, Songklanagarind Hospital, and Songkhla hospital; from June to October 2021. The questionnaires inquired about: (1) demographic information, (2) the PHQ-9 Thai version, (3) a questionnaire focusing on depressive symptoms that impacted daily life, and were originally expected to be improved due to antidepressants. All data were analyzed using descriptive statistics, and associated factors concerning depressive symptoms were analyzed by a Chi-square and a logistic regression.

**Results:**

Of all 566 respondents, the majority of them were female (75.4%). The overall mean age was 43.8 ± 18.1 years. The depressive symptoms that had high frequency, high impact on daily life, and that the participants expected that they are resolved or get better via antidepressants were: sleeping problems (81.6%), feeling depressed (79.9%), and lack of pleasure (75.4%). Most of the participants (65.7%) received one type of antidepressant, and the most prescribed antidepressants were selective serotonin reuptake inhibitors (51.1%). In regard to objectives, 45.4% of participants reported having residual depressive symptoms which included sleeping problems (71.2%), feeling down (62.6%), lack of pleasure (62.3%), and poor appetite (61.9%). The associated factors relating to residual depressive symptoms were younger age, high education level, and having physical illness.

**Conclusion:**

Almost half of patients with depression had residual symptoms, and they showed symptoms with high individual variability. Further to receiving effective treatment, a focused and individualized approach aiming for symptomatic remission, functional recovery, and quality of life improvements is key to recovery. Therefore, shared decision-making, and taking into account drug efficacy based on symptom profiles are both highly recommended.

## Background

Depression is a common and debilitating disease. It is the largest contributor to global disability. In 2012, the World Health Organization Global Burden of Disease Survey ranked depression as the fourth leading cause of disability worldwide [1]. In Thailand, for instance, the national prevalence rate of depression increased from 56 per 100,000 of population in 1997 to 197 per 100,000 of population in 2007 [2]. Depressive symptoms often start at a young age [3] and are often recurring [4–6]. According to the Diagnostic and Statistical Manual of Mental Disorders (DSM) 5-TR criteria, patients with MDD present with five (or more) of the following symptoms that have been present most of the day, for nearly every day during the same 2-week period: low mood; diminished pleasure or interest in activities; weight gain or weight loss, increase or decrease in appetite daily; hypersomnia or insomnia; psychomotor slowing or agitation; loss of energy or fatigue for nearly every day; feelings of worthlessness, excessive or inappropriate guilt; diminished ability to concentrate, make decisions or think; suicidal ideation or suicide attempts, recurrent thoughts of death [7].

MDD is described as a combination of multiple symptoms with high individual variability; and if left untreated MDD may result in progressive alterations in brain circuit function and morphometry. Recent findings suggest that pharmacotherapy may halt and possibly reverse those effects. A delay in treatment is related with poorer clinical outcomes. Therefore, the need to rapidly treat depression to full recovery should be highlighted [8]. Nowadays, over 20 antidepressants are available worldwide and they form one of the standard treatment plans for patients suffering from acute depression. They bring relief to many suffering from depressive disabilities. However, many patients with depression have residual symptoms despite showing a robust response to antidepressants [9]. They continue to have residual symptoms; causing distress, dysfunction, and an increased risk for relapse [10]. In regard to treatment by antidepressants, a prior study identified that the overall cumulative remission rate of MDD was 67% [11]. Some studies found that after 3 months of receiving antidepressants, 66% of patients were in remission and 59.5% achieved normal functioning [12]. Incomplete remission from MDD is common, with approximately one-third of patients with depression continuing to have residual symptoms during remission [13].

Concerning residual symptoms, they may meet the criteria for subsyndromal and/or minor depression [14], or having remaining symptoms after receiving treatment for at least 12 weeks [15]. In a prior study, out of all major depressive patients, 25.9% had one residual symptom, and 56.5% had two or more residual symptoms [16]. The most prevalent residual symptoms were anxiety (78%), core mood symptoms (72%), insomnia (63%), and somatic symptoms (41%) [12]. The presence of residual symptoms after an episode of MDD is related with an increased risk of recurrence and relapse [14], poor social functioning, a long-term chronic course, poor quality of life, a higher risk for suicide attempts [17], and an increasing burden on society [18]. Therefore, MDD has a destructive impact on individuals due to the nature of depressive symptoms, as a consequence of their altered functioning, and on society as a whole. These impairments could be potentially ameliorated by successful pharmacologic and psychologic treatment. Additionally, patients with MDD may show symptoms with high individual variability. Beyond the need for effective treatment, the goal ought to be symptomatic remission, the absence of residual symptoms, and the gaining of functional recovery, including a good quality of life [19, 20]; therefore, a focused individualized symptom treatment approach is necessary.

This study aimed to identify the core depressive symptoms that affected daily life and were expected to get better due to antidepressants, among Thai patients with depression. Additionally, residual symptoms, factors associated with residual symptoms, and antidepressant prescriptions were also evaluated. The identification of residual symptoms, which ones are the most frequent and in which occasions they occur, may provide support towards a more individualist intervention program based on symptom profiles.

## Methods

After being approved by the Ethics Committees of the Faculty of Medicine, Prince of Songkla University (REC: 63-522-3-1), this cross-sectional study was conducted at the two listed psychiatric outpatient clinics; Songklanagarind Hospital, which was an 800-bed university hospital serving as a tertiary referral center in Southern Thailand, and Songkhla hospital, which is a 508-bed general provincial hospital in Southern Thailand. All outpatients with depression, who had an appointment and were followed up at two psychiatric outpatient clinics; from June to October 2021, were invited to participate in the study.

Patients with the first major episode of depression, as diagnosed by their psychiatrists, were selected in the medical register based on the following criteria: ICD-10 code; F33.0-F33.9; except F33.3, aged more than 18 years, taking antidepressants for at least 12 weeks [12, 15], acknowledging their diagnosis, good understanding and use of Thai language, agreeing to participate in the study, and completing all parts of the questionnaires. Meanwhile, those who had more than one psychiatric diagnosis or comorbidity including with alcohol dependence, were unaware of their diagnosis, did not wish to participate or decided to withdraw from the study, lacked mental capacity (judged by the outpatient psychiatric nurse) to complete all of the questionnaires, were excluded.

The research assistant approached all of the eligible outpatients with depression for recruitment and handed them an information sheet, which delineated the rationale for the study and the allotted time to complete the survey. All eligible participants had at least 15–20 min to consider whether to collaborate in the study or not. To ensure that the participants’ identities would be protected, there was no requirement for their signatures. Furthermore, they were informed that their data would remain anonymous and that they could withdraw at any stage of the questionnaire without giving any reasons; and with no impact on their treatment. Participants willing to collaborate were invited to a private location to complete the questionnaires, and were informed that they could stop at any time if they felt uneasy or distressed without them needing to provide any reason. Moreover, if the participants exhibited a high level of distress or worry, advice and/or further clinical management was provided to them.

### Questionnaires

1. Personal and demographic information consisting of questions related to age, gender, religion, marital status, occupation, income, physical illness, substance usage, types, and length of time of their antidepressants prescription.
2. The Patient Health Questionnaire-9 (PHQ-9) Thai version, a self-rating questionnaire to evaluate depression consisting of nine questions. The score of each question employed a 4-point rating scale, never = 0; rarely = 1; sometime = 2; always = 3. The total score ranged from 0 to 27, with a recommended cut-off score of nine or greater which meant major depression. The questionnaire demonstrated internal consistency; Cronbach's alpha coefficient of 0.79; sensitivity of 0.53; specificity of 0.98 [21]
3. A questionnaire containing a checklist in regard to depressive symptom profiles (according to the PHQ-9, Thai version) and daily life impact, and the symptoms expected to get better due to antidepressants.
4. The questionnaire was reviewed by 3 psychiatrists; the content validity index (CVI) score was 0.8. A pilot study was conducted with 20 volunteers; thus, Cronbach’s alpha was 0.8.

### Definition

Residual depressive symptoms among patients with depression were identified in the case of patients with a first episode of depression, who received antidepressants more than 12 weeks ago [12, 15] and still had a PHQ-9 score of nine or greater.

### Statistical analysis

Descriptive statistics, such as proportions, means, standard deviations (SD), median and interquartile ranges (IQR) were calculated. Chi-square or Fisher’s exact tests and logistic regression analyses were used to identify associations between demographic characteristics, and depressive symptom profiles. The analyses were conducted using R version 3.4.1 (R Foundation for Statistical Computing). Statistical significance was defined as a p-value of less than 0.05.

## Results

### Demographic characteristics

From June to October 2021, 573 patients with depression attended both psychiatric outpatient clinics, and 566 of them agreed to participate and complete the questionnaires. The response rate was 98.8%. The mean age was 43.8 ± 18.1 years. The majority of participants were female (75.4%), Buddhist (73.7%), unmarried (56.5%), and had no history of physical illness and substance uses (56.2%, 91.7%, respectively) (Table 1). The most common physical illnesses were hypertension (31.3%), dyslipidemia (29.6%), and allergy (23.9%). The substances used by the participants were tobacco (3.4%), cannabis (1.1%), and amphetamine (0.5%). No statistically significant difference in demographic data was detected between the participants, according to the two hospitals.

**Table 1:** Demographic characteristics (*n* = 566)

Demographic characteristics	Number (%)
Gender	
Male	138 (24.4)
Female	427 (75.4)
No answer	1 (0.2)
Age (year)	
18 – 24	146 (25.8)
25–60	282 (49.8)
> 60	131 (23.1)
No answer	7 (1.2)
Marital status	
Single/divorced	320 (56.5)
Married	245 (43.3)
No answer	1 (0.2)
Religion	
Buddhism	417 (73.7)
Islam/Christianity/other	149 (26.3)
Education	
Primary school and below	138 (24.4)
Secondary school	101 (17.8)
Diploma	54 (9.5)
Bachelor’s degree and above	270 (47.7)
No answer	3 (0.5)
Occupation	
Government officer/ state enterprise employee / company employee	141 (24.9)
Self-employed / merchant/ personal business/ agriculture	166 (29.3)
Student	122 (21.6)
Unemployed	137 (24.2)
Physical illness	
No	318 (56.2)
Yes	246 (43.5)
No answer	2 (0.4)
History of substance use	
No	519 (91.7)
Yes	33 (5.8)
No answer	14 (2.5)
PHQ-9 score	
< 9	309 (54.6)
≥ 9	257 (45.4)

### Depressive symptom profiles

**Fig. 1:**
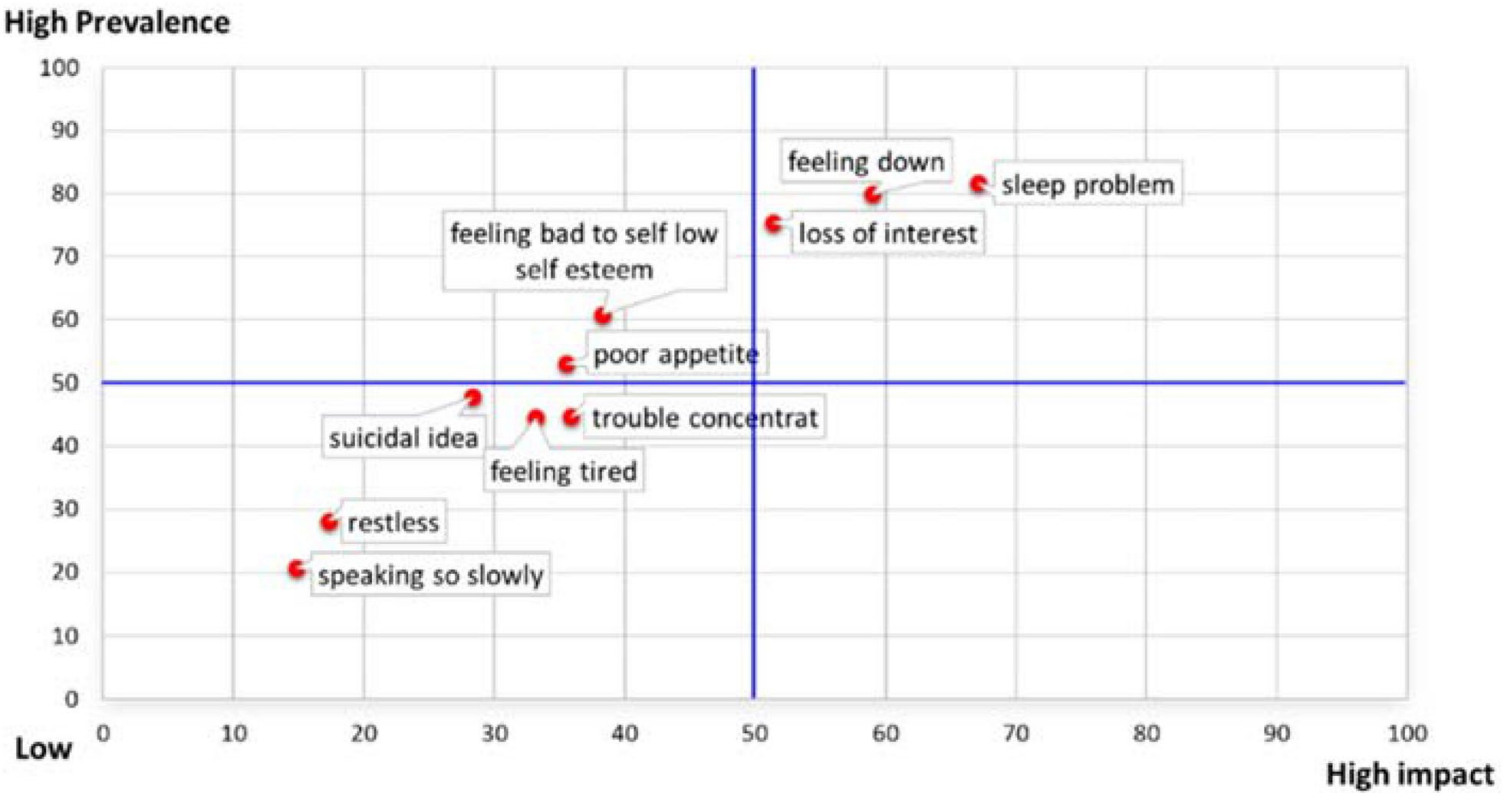
Prevalence of depressive symptoms and symptoms that impact daily life at the initial phase of the first episode of depression (*n* = 566)

In regard to depressive symptoms, the majority of participants identified core symptoms, at the initial phase of depression, that had high frequency, and high impact on patients’ daily lives and they expected that antidepressants would relieve them. These symptoms were: sleeping problems, feeling depressed or hopeless, and loss of pleasure or interest in operating things (Table 2, Fig. 1).

**Table 2:** Core depressive symptoms, symptom impact on daily life and symptoms that are expected to be relieved (more than 1 answer) (n = 566)

Item	Number (%)		
	Depressive symptom	Symptom impact daily life	Expectation of antidepressant relieve symptom
Trouble falling or staying asleep, or sleep too much	462 (81.6)	380 (67.1)	350 (61.8)
Feeling down, depressed or hopeless	452 (79.9)	334 (59.0)	319 (56.4)
Loss of interest or pleasure in doing things	427 (75.4)	291 (51.4)	251 (44.3)
Feeling bad about myself or failure or have let myself or my family down	344 (60.8)	217 (38.3)	203 (35.9)
Poor appetite or overeating	300 (53.0)	201 (35.5)	133 (23.5)
Thoughts that you would be better off dead, or of hurting myself in some way	271 (47.9)	160 (28.3)	147 (26.0)
Trouble concentrating on things, diminished ability to think	253 (44.7)	203 (35.9)	167 (29.5)
Feeling tired or having little energy	252 (44.5)	188 (33.2)	137 (24.2)
Restless that moving around a lot more than usual	159 (28.1)	98 (17.3)	71 (12.5)
Moving or speaking so slowly that other people could have noticed	117 (20.7)	84 (14.8)	64 (11.3)

**Fig. 2:**
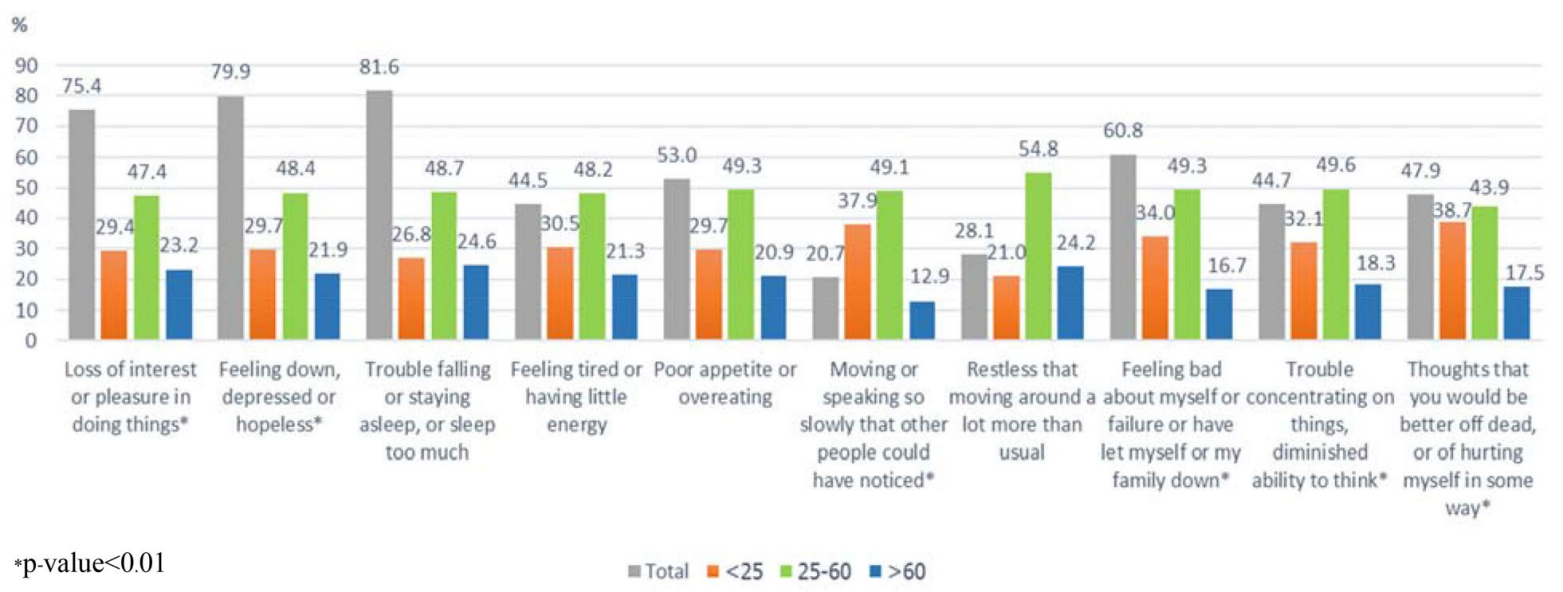
Depressive symptoms at the initial phase of the first episode of depression as per age group (*n* = 566)

In regard to age, there was a statistically significant difference in depressive symptoms at the initial phase of illness between different age groups (*p* < 0.001). The most common depressive symptoms among young adults were thoughts of being better off dead or of hurting themselves (38.7%), moving or speaking too slowly (37.9%), feeling bad about themselves or failure (34%), trouble concentrating on things, and a diminished ability to think (32.1%), while the main depressive symptoms among the elderly were sleeping problems (24.6%), restlessness (24.2%), and a loss of interest or pleasure (23.2%) (Fig. 2).

### Residual depressive symptoms

**Fig. 3:**
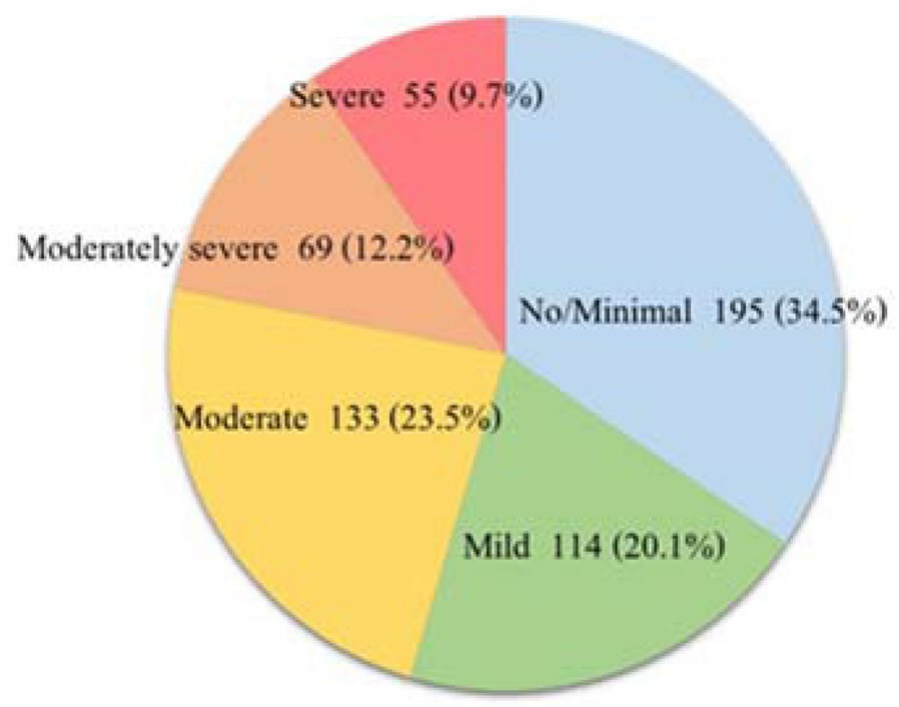
Depression according to PHQ-9 (*n* = 566)

Of all participants, 257 (45.4%) reported having a PHQ-9 score of nine or greater, indicating the presence of residual depressive symptoms (Table 1). Moreover, 55 (9.7%) participants were still being severely depressed (Fig. 3).

**Fig. 4:**
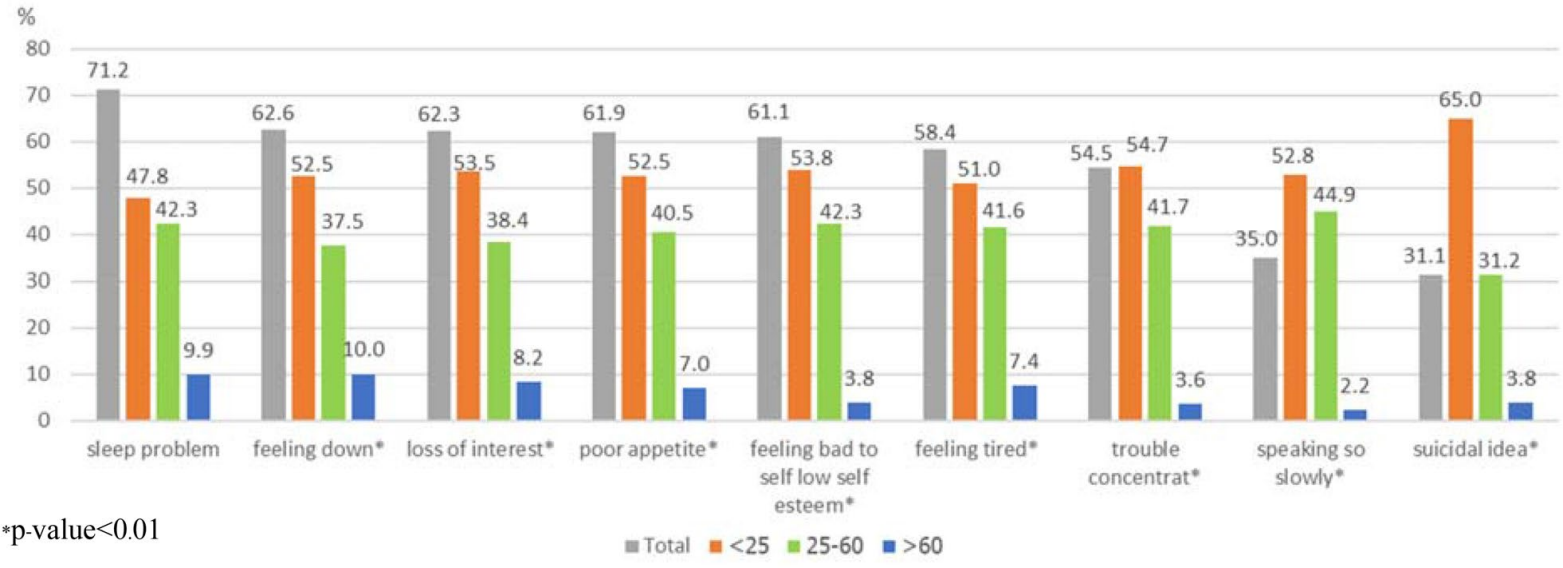
Frequency of residual depressive symptoms as per age group (*n* = 257)

**Fig. 5:**
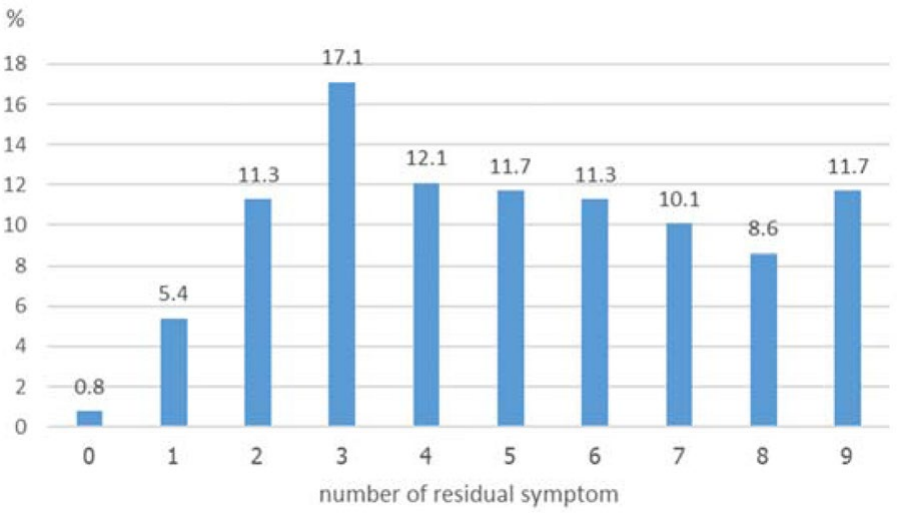
Number of residual depressive symptoms (*n* = 257)

The most common residual depressive symptoms were sleeping problems (71.2%), low mood (62.6%), loss of interest or pleasure (62.3%), and poor appetite (61.9%). In regard to age groups, the most common residual depressive symptoms among young adults were thoughts of hurting themselves or suicidal ideation (65%), trouble in concentration (54.7%), feeling bad or low self-esteem (53.8%), and loss of interest (53.5%), while the main residual depressive symptoms among the elderly were low mood (10%), and sleeping problems (9.9%) (Fig. 4). Furthermore, 44 (17.1%) participants had three residual depressive symptoms, and 8.6% to 12.1% of participants had more than three residual depressive symptoms (Fig. 5).

### Treatment profile

One hundred and thirty-seven (24.2%) participants received antidepressants for less than one year, and half of them received antidepressants for more than 2 years (Table 3). The median duration of receiving antidepressants was 26.9 months (IQR = 12.0, 62.9).

**Table 3:** Demographic characteristic and treatment categorized by PHQ-9 score

Variables		Number (%) PHQ-9		*P*-value
	Total (*n* = 566)	< 9 (*n* = 309)	≥ 9 (*n* = 257)	
Gender				0.068
Male	138 (24.4)	85 (27.6)	53 (20.6)	
Female	427 (75.4)	223 (72.4)	204 (79.4)	
No answer	1 (0.2)			
Age (year)				< 0.001
18–24	146 (25.8)	35 (11.6)	111 (43.4)	
25–60	282 (49.8)	165 (54.5)	117 (45.7)	
≥ 60	131 (23.1)	103 (34)	28 (10.9)	
No answer	7 (1.2)			
Marital status				< 0.001
Single/divorced	320 (56.5)	145 (47.1)	175 (68.1)	
Married	245 (43.3)	163 (52.9)	82 (31.9)	
No answer	1 (0.2)			
Religion				0.038
Buddhism	417 (73.7)	239 (77.3)	178 (69.3)	
Islam / Christianity / others	149 (26.3)	70 (22.7)	79 (30.7)	
Education				< 0.001
Primary/secondary school and below	239 (42.2)	161 (52.3)	78 (30.6)	
Diploma / bachelor’s degree and above	324 (57.2)	147 (47.7)	177 (69.4)	
No answer	3 (0.5)			
Occupation				< 0.001
Government officer/ state enterprise employee / company employee	141 (24.9)	77 (24.9)	64 (24.9)	
Self-employed / merchant/ personal business/ agriculture	166 (29.3)	107 (34.6)	59 (23)	
Student	122 (21.6)	30 (9.7)	92 (35.8)	
Unemployed	137 (24.2)	95 (30.7)	42 (16.3)	
Physical illness				0.143
No	318 (56.2)	164 (53.4)	154 (59.9)	
Yes	246 (43.5)	143 (46.6)	103 (40.1)	
No answer	2 (0.4)			
History of substance use				0.605
No	519 (91.7)	284 (94.7)	235 (93.3)	
Yes	33 (5.8)	16 (5.3)	17 (6.7)	
No answer	14 (2.5)			
Duration of received antidepressants				< 0.001
> 3–6 months	86 (15.2)	31 (10.3)	55 (21.8)	
> 6 months-1 years	51 (9.0)	28 (9.3)	23 (9.1)	
> 1–2 years	135 (23.9)	66 (21.9)	69 (27.3)	
> 2–3 years	75 (13.3)	43 (14.2)	32 (12.6)	
> 3–5 years	67 (11.8)	39 (12.9)	28 (11.1)	
> 5 years	141 (24.9)	95 (31.5)	46 (18.2)	
No answer	11 (1.9)			
Duration of received antidepressants (month):				< 0.001
Mean (IQR)	26.9 (12.0, 62.9)	36 (16.0, 107.7)	24 (8.0, 48.0)	
Number of antidepressants				0.596
One	372 (65.7)	208 (67.3)	164 (63.8)	
Two	162 (28.6)	83 (26.9)	79 (30.7)	
Three or more	32 (5.7)	18 (5.8)	14 (5.4)	

Out of all participants, 372 (65.7%) and 162 (28.6%) received one and two types of antidepressants, respectively. The most prescribed antidepressants were selective serotonin reuptake inhibitors (SSRIs) (51.1%); sertraline (27.9%); fluoxetine (15%), and SSRIs plus other antidepressants (13.9%) (Table 4).

**Table 4:** Type of antidepressants that the patients received (*n* = 566**)**

Type of antidepressants	Number (%)
One type of antidepressants	372 (65.7)
TCAs	15 (2.6)
Nortriptyline	8 (1.4)
Amitriptyline	3 (0.5)
Imipramine	3 (0.5)
Clomipramine	1 (0.2)
SSRIs	289 (51.1)
Sertraline	158 (27.9)
Fluoxetine	85 (15.0)
Escitalopram	27 (4.8)
Fluvoxamine	19 (3.4)
Other	68 (12.1)
Agomelatine	16 (2.9)
Desvenlafaxine	13 (2.3)
Mirtazapine	12 (2.1)
Vortioxetine	9 (1.6)
Mianserin	8 (1.4)
Bupropion	8 (1.4)
Duloxetine	2 (0.4)
Two types of antidepressants	162 (28.6)
TCAs + SSRIs	34 (6.0)
TCAs + other	6 (1.1)
SSRIs + SSRIs	14 (2.5)
SSRIs + other	78 (13.9)
Other + other	30 (5.3)
Three types of antidepressants or more	32 (5.7)

### The association between demographic characteristics, treatment profiles and residual depressive symptoms

Variables whose *p*-values from the univariate analysis were lower than 0.2 were included in the initial model for multivariate analysis. Multivariate analysis indicated that age, religion, education, and physical illness were statistically significant factors associated with having residual depressive symptoms. The patients with depression aged between 18 to 24, had a higher rate of residual depressive symptoms than the older group, the adjusted odds ratio (AOR) was 12.08, 95% confidence interval (CI) at 6.28 to 23.23. The same was true when comparing them with those whose religion was Islam or Christianity, had higher education, and physical illness; AOR (95% CI) was 1.70 (1.10, 2.62), 1.73 (1.16, 2.57), and 1.55 (1.02, 2.35), respectively (Table 5).

**Table 5:** Factors associated with residual depressive symptoms

Factors	Crude OR (95%CI)	Adjusted OR (95% CI)	*P*-value *LR*-test
Age (year)			< 0.001
> 60	Reference	Reference	
18–24	11.86 (6.68, 21.05)	12.08 (6.28, 23.23)	
25–60	2.59 (1.59, 4.21)	2.48 (1.46, 4.20)	
Religion			0.016
Buddhism	Reference	Reference	
Islam/Christianity/others	1.62 (1.11, 2.38)	1.70 (1.10, 2.62)	
Education			0.007
Primary/secondary school and below	Reference	Reference	
Diploma / bachelor’s degree and below	2.35 (1.66, 3.35)	1.73 (1.16, 2.57)	
Physical illness			0.036
No	Reference	Reference	
Yes	0.76 (0.54, 1.06)	1.55 (1.02, 2.35)	

### Discussion

This study indicated that the most common depressive symptoms and symptoms that have a strong impact on a patient's daily life were sleeping problems, feeling depressed or hopeless, and the loss of pleasure in doing things. There was a statistically significant difference in common depressive symptoms between age groups. Selective serotonin reuptake inhibitors (SSRIs), serotonin and norepinephrine reuptake inhibitors (SNRIs), mirtazapine, bupropion, and agomelatine being first-line recommendations for pharmacotherapy for MDD [15, 22]. Furthermore, the most common antidepressant prescription by our psychiatrists was SSRIs (51.1%). Notably, 45.4% of participants reported having residual depressive symptoms (PHQ-9 ≥ 9). The most common residual depressive symptoms were sleep problems (71.2%), feeling depressed (62.6%), loss of pleasure (62.3%), and poor appetite (61.9%). Moreover, the associated factors relating to residual depressive symptoms were younger age, high education level, and having physical illness.

In regard to depressive symptoms profiles, in this study, we found that sleeping problems, feeling depressed or hopeless, and loss of interest or pleasure were the symptoms presenting with the highest frequency and level of impact on the patients’ daily lives. The depressive symptom profiles from our study feature a number of differences versus a study from Canada [22] which identified that fatigue, poor concentration or diminished ability to think, loss of interest or pleasure, low mood, and feeling worthlessness or guilt were the symptoms with the highest frequency and level of impact on patients’ daily lives. A potential explanation for these discrepancies may be due to different study instruments, population ethnicity, age group, and cultures. However, our study reported a statistically significant difference in depressive symptoms between young adults, adults, and elderly age groups. These results were similar to a prior study that identified symptom differences among young adults, adults, and elderly patients with depression. In regard to young adults, the previous study found that physical or vegetative disturbances (changes in appetite, weight, loss of energy and sleep changes) were common. Moreover, a vegetative symptom profile was only seen in young adults with depression. In regard to adults, concentration difficulties, and anhedonia/loss of interest were more common. [23]. Therefore, before prescription, physicians should consider the core depressive symptoms which should be particularly targeted; and that this may vary due to age group symptom related differences.

Considering residual depressive symptoms, almost half of the participants (45.4%) reported residual depressive symptoms which included sleeping problems, feeling down, loss of pleasure, and poor appetite. Additionally, this study identified that the mean duration of receiving antidepressants was at 26.9 months, and that 65.7% of participants received one type of antidepressant, and that SSRIs were the most common antidepressants prescribed. In regard to the neurotransmitter model of function in depression, a well-known concept, depression is described as a combination of two components: a lack of positive affect and an increase in negative affect. Negative affect means viewing the world as an unpleasant, disturbing, hostile, and threatening place. Lack of positive affect equals an inability to take pleasure rewards from normal activities. During treatment with antidepressant medication, some patients might experience particularly unresponsive depression-related symptoms with a higher lack of positive affect, while other patients might experience depression with an increase of negative affect, such as symptoms of anxiety [24]. As per a prior study, clinical trials of antidepressants have shown that some dual-acting antidepressants, such as serotonin–norepinephrine reuptake inhibitors, may result in higher rates of remission than other pharmacological agents, and with fewer residual depressive symptoms than treatment with only SSRIs [16]. Based on recent information from studies about antidepressants, it might be possible to assign specific symptoms of depression to specific neurochemical mechanisms. Norepinephrine may be related to energy and alertness as well as attention, interest in life, and anxiety; serotonin to obsessions, and compulsion, anxiety; and dopamine to having an interest in life, pleasure and reward, as well as motivation. Increasing any of these three neurotransmitters could elevate mood, but the other elements of depression may be particularly responsive to a specific neurotransmitter [25]. Therefore, the different neurotransmitters may regulate different brain functions in patients with depression; different antidepressants due to their dissimilar pharmacology target a diversity of neurotransmitters, and these may affect different symptoms of depression. Knowing which particular neurotransmitters are related with what symptoms of MDD may help physicians prescribe pharmacological agents that target specific mechanisms that in turn target specific depressive symptoms [26, 27]. In this study, most participants received one class of antidepressant, SSRIs, and this may be the reason for the lower rates of remission versus treatment with multi-acting antidepressants. In addition, the most common residual depressive symptoms were sleeping problems, loss of pleasure, and poor appetite; and it might be possible that these specific symptoms of depression did not respond as well to SSRIs. It is recommended that physicians should be concerned by the individual variability of symptoms and ensure that selecting antidepressants targets core depressive symptoms in a specific manner. Additionally, sleeping problems may be a comorbid disease, such as insomnia disorder [28]. Therefore, monitoring residual depressive symptoms in the remission phase may be necessary.

In this study, patients with depression, aged 18 to 24, had a higher rate of residual depressive symptoms than the older group, the AOR was 12.08. Based on prior studies, depression was a prevalent and serious mental disorder among youth adults or adolescents and adults, and it was related with suicide, an increase in family problems, substance abuse, absenteeism [29], and disability that could be lowered to perform life activities associated to work performance and/or academic achievement including a decrease in student grade point average (GPA) [24]. Furthermore, the pattern of increasing interference of depressive symptoms with academic performance, might peak during the month of diagnosis and decrease afterward, with the lowest levels reported four to six months post-diagnosis [30]. Thus, antidepressants that specifically target the depressive symptom of youth-adult patients should be prescribed rapidly and effectively.

Additionally, this study identified that patients with higher education and experiencing a physical illness had a higher rate of residual depressive symptoms than the rest of the group. Higher levels of education could be associated with them having a higher level of work responsibilities and as a result more tension. While having physical illness may be associated with elevated life stress. Some physical symptoms could also produce anxiety, distress, suffering, and sleeping problems. Therefore, these factors may make depression more complicated.

Finally, several other factors that were not addressed by our study could have possibly influenced the response to treatment and the presence of residual depressive symptoms, such as alcohol usage, and biological factors; an imbalance of pteridine metabolism in depression [31]. The prior study reported increased levels of markers of inflammation and oxidative stress in MDD. Moreover, likewise poorer antidepressant treatment response was related to higher baseline levels of the major oxidative stress marker, F2-isoprostanes, in vivo [32]. However, when treating patients with depression in clinical settings, physicians should deliberately select an antidepressant based on the specific presenting and individual symptom profiles of the patient. Selecting the appropriate antidepressant for a patient’s particular symptoms might offer the best chance for a successful response to treatment [25, 26]. Furthermore, the importance of shared decision-making, drug efficacy, side effects, medical treatment rights, covered medical expenses, and economic status should also be taken into consideration. Therefore, psychiatric training and national mental health care policy should be concerned with the desire for treatment of depression related to high individual variability.

This study had both limitations and strengths worth mentioning. To our knowledge, this is the only study on this topic conducted in southern Thailand during the last decade. However, this study had some limitations as it was a cross-sectional survey and utilized self-administered questionnaires; therefore, some misunderstandings about the intended meaning of the questions may have taken place. Another drawback is that our data were quantitative, the sample size and that the participants were restricted to patients with depression in lower, southern Thailand. Most participants were female in gender and had a high educational level. Hence, these results may not demonstrate the situation or condition of patients with depression in all gender groups, all educational levels, or the whole country in a proportionate manner. It is recommended that future studies feature a larger number of patients with depression from other hospitals in Thailand; and that a more comprehensive, multi-centered research study, with more qualitative or in-depth methods should be employed. Moreover, there are some factors to be aware of, such as alcohol usage or abuse, medication adherence, drug tolerability, psychotherapy, family, and social support, as these may influence the prognosis or the outcome of treatment among patients with depression.

## Conclusion

Almost half of the patients with depression had residual symptoms, and they showed symptoms with high individual variability. Further to receiving effective treatment, a focused and individualized approach aimed to symptomatic remission, and functional recovery is necessary. Therefore, shared decision-making, and taking into account drug efficacy based on individual symptom profiles are both highly recommended.

## Data Availability

The qualitative data used in and analyzed during the current study cannot be made publicly available for confidentiality reasons, but they can be available on request from the corresponding author.

## Abbreviations

MDD: Major depressive disorder
PHQ-9: Patient Health Questionnaire-9
SNRIs: Serotonin and norepinephrine reuptake inhibitors
SSRIs: Selective serotonin reuptake inhibitors

## References

[1] Ferrari AJ, Somerville AJ, Baxter AJ, Norman R, Patten SB, Vos T (2013). Global variation in the prevalence and incidence of major depressive disorder: a systematic review of the epidemiological literature. Psychol Med.

[2] Songprakun W, McCann TV (2012). Effectiveness of a self-help manual on the promotion of resilience in individuals with depression in Thailand: a randomised controlled trial. BMC Psychiatry.

[3] Thapar A, Collishaw S, Pine DS, Thapar AK (2012). Depression in adolescence. Lancet.

[4] Limosin F, Loze JY, Zylberman-Bouhassira M, Schmidt ME, Perrin E, Rouillon F (2004). The course of depressive illness in general practice. Can J Psychiatr.

[5] Yiend J, Paykel E, Merritt R, Lester K, Doll H, Burns T (2009). Long term outcome of primary care depression. J Affect Disord.

[6] Katon W, Rutter C, Ludman EJ, Von Korff M, Lin E, Simon G (2001). A randomized trial of relapse prevention of depression in primary care. Arch Gen Psychiatr.

[7] American Psychiatric Association. Major depressive disorder. 2022:30–39. https://psychiatry.org/File%20Library/Psychiatrists/Practice/DSM/DSM-5-TR/APA-DSM5TR-MajorDepressiveDisorder.pdf. Accessed 18 Oct 2022.

[8] Oluboka OJ, Katzman MA, Habert J, McIntosh D, MacQueen GM, Milev RV (2018). Functional recovery in major depressive disorder: providing early optimal treatment for the individual patient. Int J Neuropsychopharmacol.

[9] Lee AS (2003). Better outcomes for depressive disorders?. Psychol Med.

[10] Pintor L, Torres X, Navarro V, Matrai S, Gasto C (2004). Is the type of remission after a major depressive episode an important risk factor to relapses in a 4-year follow up?. J Affect Disord.

[11] Rush AJ, Trivedi MH, Wisniewski SR, Nierenberg AA, Stewart JW, Warden D (2006). Acute and longer-term outcomes in depressed outpatients requiring one or several treatment steps: a STAR*D report. Am J Psychiatr.

[12] Romera I, Perez V, Ciudad A, Caballero L, Roca M, Polavieja P (2013). Residual symptoms and functioning in depression, does the type of residual symptom matter?. A post-hoc analysis BMC Psychiatr.

[13] Kennedy N, Foy K (2005). The impact of residual symptoms on outcome of major depression. Curr Psychiatr Rep.

[14] Nierenberg AA, Wright EC (1999). Evolution of remission as the new standard in the treatment of depression. J Clin Psychiatr.

[15] Kudlow PA, McIntyre RS, Lam RW (2014). Early switching strategies in antidepressant non-responders: current evidence and future research directions. CNS Drugs.

[16] Nierenberg AA, Keefe BR, Leslie VC, Alpert JE, Pava JA, Worthington JJ (1999). Residual symptoms in depressed patients who respond acutely to fluoxetine. J Clin Psychiatr.

[17] Hiranyatheb T, Nakawiro D, Wongpakaran T, Wongpakaran N, Bookkamana P, Pinyopornpanish M (2016). The impact of residual symptoms on relapse and quality of life among Thai depressive patients. Neuropsychiatr Dis Treat.

[18] Wittchen HU, Jacobi F, Rehm J, Gustavsson A, Svensson M, Jonsson B (2011). The size and burden of mental disorders and other disorders of the brain in Europe 2010. Eur Neuropsychopharmacol.

[19] Zimmermann TM, Clouth J, Elosge M, Heurich M, Schneider E, Wilhelm S (2013). Patient preferences for outcomes of depression treatment in Germany: a choice-based conjoint analysis study. J Affect Disord.

[20] Johnson MD, Varano CA, Hay J, Unutzer J, Hinton L (2013). Depression treatment preferences of older white and Mexican origin men. Gen Hosp Psychiatr.

[21] Lotrakul M, Sumrithe S, Saipanish R (2008). Reliability and validity of the Thai version of the PHQ-9. BMC Psychiatry.

[22] Kennedy SH, Lam RW, McIntyre RS, Tourjman SV, Bhat V, Blier P (2016). Canadian network for mood and anxiety treatments (CANMAT) 2016 clinical guidelines for the management of adults with major depressive disorder: section 3. Pharmacol treatments Can J Psychiatr.

[23] Rice F, Riglin L, Lomax T, Souter E, Potter R, Smith DJ (2019). Adolescent and adult differences in major depression symptom profiles. J Affect Disord.

[24] Greenberg PE, Stiglin LE, Finkelstein SN, Berndt ER (1993). The economic burden of depression in 1990. J Clin Psychiatr.

[25] Moret C, Briley N (2011). The importance of norepinephrine in depre7ssion. Neuropsychiatr Dis Treat.

[26] Nutt DJ (2008). Relationship of neurotransmitters to the symptoms of major depressive disorder. J Clin Psychiatr.

[27] Nutt DJ (2006). The role of dopamine and norepinephrine in depression and antidepressant treatment. J Clin Psychiatr.

[28] Staner L (2010). Comorbidity of insomnia and depression. Sleep Med Rev.

[29] Emslie GJ, Mayes TL (1999). Depression in children and adolescents: a guide to diagnosis and treatment. CNS Drugs.

[30] Hysenbegasi A, Hass SL, Rowland CR (2005). The impact of depression on the academic productivity of university students. J Ment Health Policy Econ.

[31] Cavaleri D, Bartoli F, Capogrosso CA, Guzzi P, Moretti F, Riboldi I (2023). Blood concentrations of neopterin and biopterin in subjects with depression: a systematic review and meta-analysis. Prog Neuropsychopharmacol Biol Psychiatr.

[32] Lindqvist D, Dhabhar FS, James SJ, Hough CM, Jain FA, Bersani FS (2017). Oxidative stress, inflammation and treatment response in major depression. Psychoneuroendocrinology.

